# Correction: White-tailed deer population declines in a high-prevalence chronic wasting disease region of Arkansas, USA

**DOI:** 10.1371/journal.pone.0359536

**Published:** 2026-09-28

**Authors:** Heather E. Gaya, Marcelo H. Jorge, Lisa A. Jorge, Mark G. Ruder, Gino J. D’Angelo, Richard B. Chandler, Michael J. Chamberlain

In the Discussion, there is an error in the first four sentence of the second paragraph. The correct sentence are: To the best of our knowledge, no previous published studies have estimated deer densities in or around the Arkansas northern CWD management zone.

In the Discussion, there is an error in the fourth sentence of the third paragraph. The correct sentence is: In addition to environmental factors, predation by coyotes (Canis latrans) or black bears (Ursus americanus) may have contributed to high fawn mortality and lower population growth rates [44,45], though no studies have documented a substantial increase in predator populations in this area during the study period.

There is an error in reference 36. The correct reference is: Peitz DG, Haach-Gaynor JL. White-tailed deer monitoring at Arkansas post national memorial, Arkansas: 2005–2024 trend report [Internet]. Fort Collins, Colorado: National Park Service; Report No.: Science Report NPS/SR-2024/215. Available from: https://doi.org/10.36967/2306678.
