# Supplementary material for: White-tailed deer population declines in a high-prevalence chronic wasting disease region of Arkansas, USA
Source: PLoS One. 2026 Jan 7;21(1):e0340070. doi: 10.1371/journal.pone.0340070 (PMC12779150; doi:10.1371/journal.pone.0340070)
Supplement: S1 Table — (DOCX) [file pone.0340070.s001.docx]

Supplemental information for: White-tailed deer population declines in a high-prevalence chronic wasting disease region of Arkansas, USA

**Table S1. Estimates of encounter rate and spatial scale parameters.**

| Parameter | Site | Stage | 2021 | 2022 | 2023 | 2024 |
| --- | --- | --- | --- | --- | --- | --- |
| $\lambda_{0}$ | Erbie | Female | 0.03  (0.01 – 0.10) | 0.18  (0.07 – 0.38) | 0.16  (0.07 – 0.34) | 0.16  (0.07 – 0.29) |
|  |  | Male | 0.03  (0.01 – 0.11) | 0.02  (0.004 – 0.06) | 0.02  (0.004 – 0.08) | 0.02  (0.002 – 0.15) |
|  | Gene Rush | Female | 0.07  (0.04 – 0.13) | 0.02  (0.01 – 0.06) | 0.02  (0.01 – 0.05) | 0.17  (0.10 – 0.29) |
|  |  | Male | 0.03  (0.01 – 0.07) | 0.01  (0.001– 0.04) | 0.004  (0.001 – 0.01) | 0.01  (0.009 – 0.06) |
|  | Tyler Bend | Female | 0.08  (0.05 – 0.14) | 0.05  (0.02 – 0.09) | 0.04  (0.02 – 0.07) | 0.10  (0.05 – 0.18) |
|  |  | Male | 0.01  (0.001 – 0.02) | 0.01  (0.001 – 0.02) | 0.02  (0.003 – 0.09) | 0.01  (0.002 – 0.04) |
| $\sigma$ | Erbie | Female | 0.32  (0.25 – 0.41) | 0.15  (0.13 – 0.17) | 0.20  (0.18 – 0.23) | 0.20  (0.18 – 0.22) |
|  |  | Male | 0.36  (0.31 – 0.43) | 0.41  (0.36 – 0.49) | 0.33  (0.26 – 0.44) | 0.41  (0.32 – 0.54) |
|  | Gene Rush | Female | 0.19  (0.18 – 0.20) | 0.18  (0.17 – 0.19) | 0.22  (0.20 – 0.23) | 0.20  (0.18 – 0.21) |
|  |  | Male | 0.29  (0.26 – 0.32) | 0.23  (0.20 – 0.26) | 0.78  (0.70 – 0.88) | 0.22  (0.19 – 0.25) |
|  | Tyler Bend | Female | 0.20  (0.19 – 0.22) | 0.20  (0.19 – 0.22) | 0.26  (0.24 – 0.29) | 0.14  (0.13 – 0.15) |
|  |  | Male | 0.34  (0.30 – 0.39) | 0.21  (0.19 – 0.23) | 0.18  (0.15 – 0.21) | 0.30  (0.26 – 0.35) |

Estimates of encounter rate and spatial scale parameters for a spatial mark resight model of white-tailed deer in a high CWD prevalence region of northern Arkansas from July 1 – July 14, 2021 to 2024. Estimates describe the median and 95% credible interval. Spatial scale parameters are reported in kilometers.
