# Supplementary material for: White-tailed deer population declines in a high-prevalence chronic wasting disease region of Arkansas, USA
Source: PLoS One. 2026 Jan 7;21(1):e0340070. doi: 10.1371/journal.pone.0340070 (PMC12779150; doi:10.1371/journal.pone.0340070)
Supplement: S1 Fig — (DOCX) [file pone.0340070.s002.docx]

Supplemental information for: White-tailed deer population declines in a high-prevalence chronic wasting disease region of Arkansas, USA


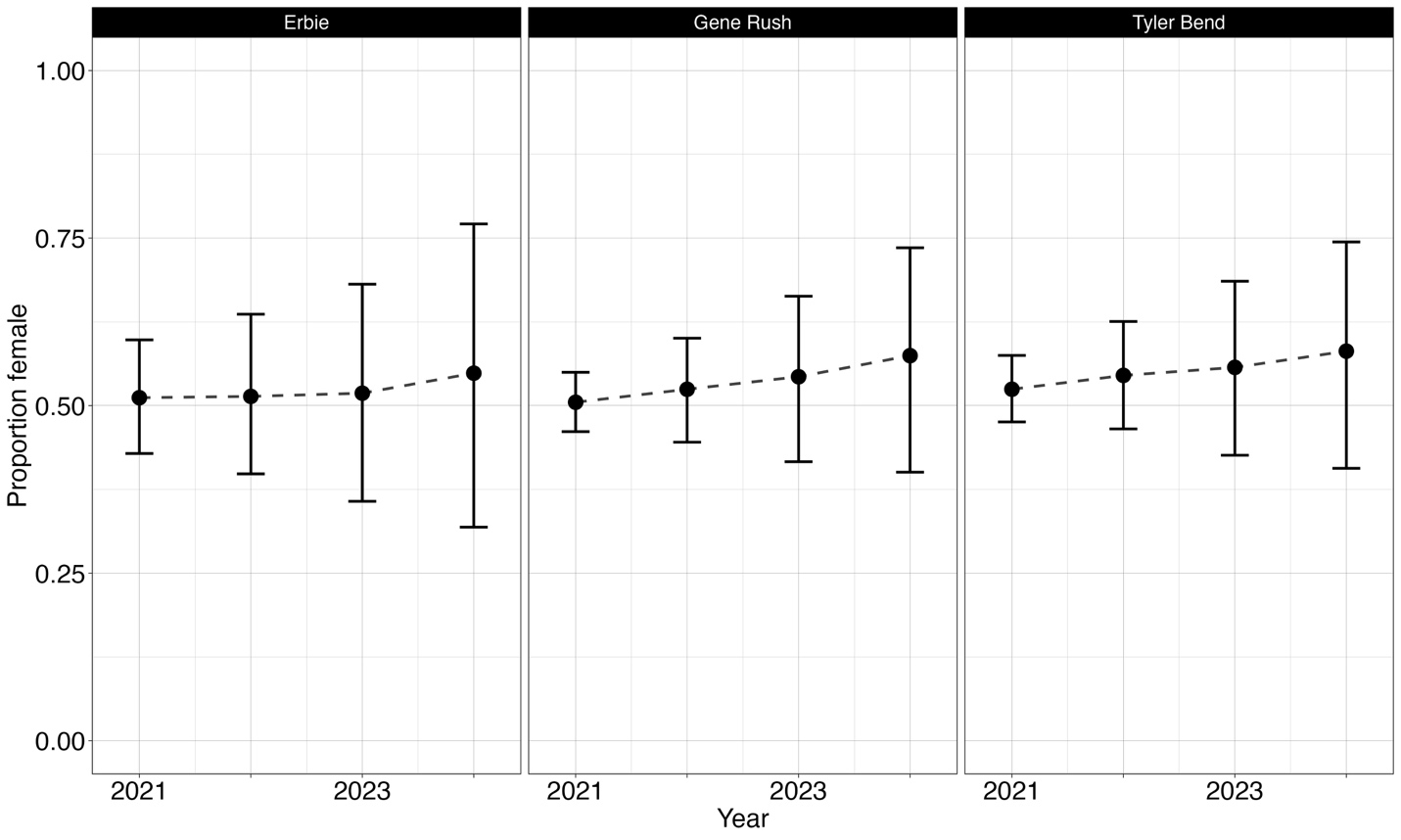


Figure S1. Estimated sex ratio of adult white-tailed deer for 3 sites in Arkansas from 2020 to 2024. Error bars depict 95% credible intervals.
