# Supplementary material for: White-tailed deer population declines in a high-prevalence chronic wasting disease region of Arkansas, USA
Source: PLoS One. 2026 Jan 7;21(1):e0340070. doi: 10.1371/journal.pone.0340070 (PMC12779150; doi:10.1371/journal.pone.0340070)
Supplement: S2 Fig — (DOCX) [file pone.0340070.s003.docx]

Supplemental information for: White-tailed deer population declines in a high-prevalence chronic wasting disease region of Arkansas, USA


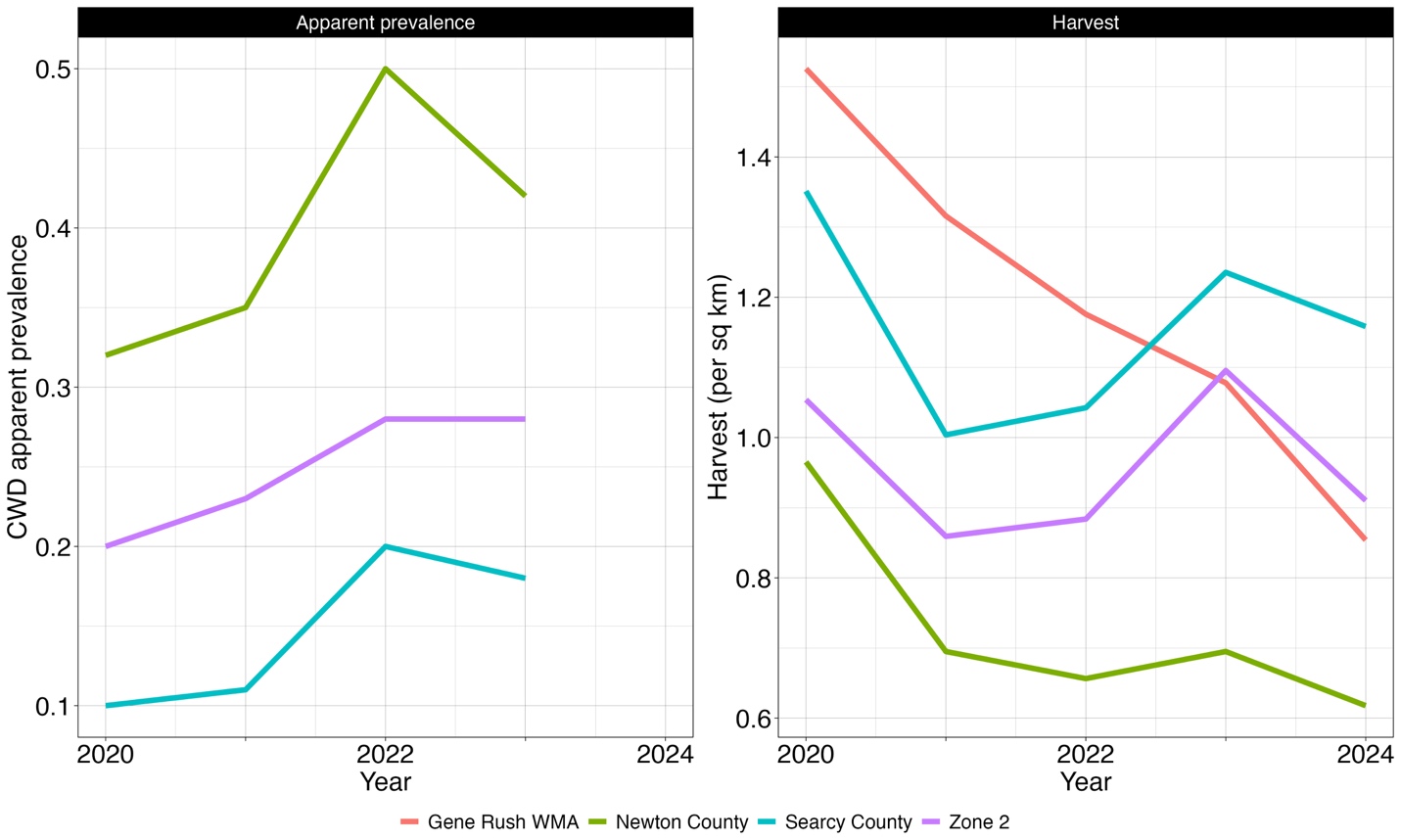


Figure S2. Apparent prevalence and harvest rates of white-tailed deer for 4 areas in Arkansas from 2020 to 2024. Data sourced from the Arkansas Chronic Wasting Disease Management and Response 2023-2024 annual report published by the Arkansas Game and Fish Commission^1–3^.

Citations:

1. Arkansas Game and Fish Commission. *Arkansas Chronic Wasting Disease Management and Response, 2023 -24 Annual Report*. https://drive.google.com/file/d/1G4ePbX0Ofl8iaHpbX-HLIVQJb_sJYpeY/view (2024).

2. Arkansas Game and Fish Commission. *Arkansas Chronic Wasting Disease Management and Response, 2024 -25 Annual Report*. https://drive.google.com/file/d/1VC0TjxU7JkMbzPC_YsWwt8qzjzCZterz (2025).

3. Arkansas Game and Fish Commission. CWD in Arkansas. https://www.agfc.com/hunting/deer/chronic-wasting-disease/cwd-in-arkansas/ (2025).
