# Supplementary material for: White-tailed deer population declines in a high-prevalence chronic wasting disease region of Arkansas, USA
Source: PLoS One. 2026 Jan 7;21(1):e0340070. doi: 10.1371/journal.pone.0340070 (PMC12779150; doi:10.1371/journal.pone.0340070)
Supplement: Attachment — Submitted filename: Response_to_Review.docx [file pone.0340070.s005.docx]

Dear Dr. Caughey,

Thank you for giving us the opportunity to submit a revised draft of our manuscript, White-tailed deer population declines in a high-prevalence chronic wasting disease region of Arkansas, USA (Submission ID JWM-25-0070). We appreciate the insightful comments on our paper and have incorporated many of the suggested changes into our revised document.

In addition to responding to specific reviewer comments, we re-ran our analysis to include a time trend in the abundance estimates. This allowed us to more accurately quantify the decline in deer populations and separate trends between males and females, while also improving convergence and producing more precise estimates.

Below, we detail our responses (normal font) to the reviewer comments ***(bold italics).***

***Reviewer 1 Summary Review:
This study uses a novel approach for estimating deer density and evaluating changes in density associated with CWD prevalence/history. This is an important topic as it attempts to estimate the impact of a complex disease (CWD) on a socially and economically important wildlife species (white-tailed deer). I appreciate the extensive efforts, time, and resources required for this study. There is an extensive description of the SCR methodology used to estimate annual deer density at 3 different study sites over a 4-year period. This may be the first study to estimate the association between CWD and deer density. Although I am not a SCR expert I found this analysis to be confusing and not clearly described. I doubt that many readers would be able to replicate the analyses as described in the paper. I strongly recommend a diagram to show more clearly how the various model pieces integrate to estimate deer density and clear/consistent definition of model parameters (including units).***

We thank the reviewer for his comments and agree that our analysis was not written so that readers unfamiliar with SCR would be able to clearly follow our methods. We have made extensive edits to the methods section and rerun our analysis to include a time trend that allows us to discuss the trends in deer abundance in more quantitative terms.

***Despite the extensive analysis it appears that density estimates are highly imprecise and are much lower than would be expected based on external deer abundance estimates and on harvest from these areas. Both these factors make me reluctant to accept the study conclusions of an association between deer density and CWD prevalence.***

As mentioned above, we have improved on our previous analysis so the estimates are more precise. We have also adjusted several areas in the text that reported deer harvest in square miles, which made our estimate of deer densities in square kilometers seem illogically low.

***I found that the majority of the paper focused on methods used to estimate deer density while the spatial distribution/prevalence of CWD was largely ignored. The paper makes unsupported (assumed) spatial patterns of CWD distribution. In addition, CWD prevalence at the study sites is only vaguely reported using ranges of prevalence. The paper concludes, without scientific/statistical analysis, that deer density declined during the study. Given the lack of precision in estimated deer density I am skeptical such a conclusion is valid. Unless the authors can substantially improve the precision (and hopefully negative bias) of the density estimates I doubt a robust evaluation of density and CWD are possible. I recommend a much more rigorous evaluation of the landscape distribution and prevalence of CWD.***

We appreciate the reviewer’s concerns and have added in additional discussion material to address the use of a proxy for CWD prevalence. We have also included information from AGFC’s yearly harvest reports in a supplemental figure that show the CWD sample prevalence in the study area counties from 2020- 2024. While Arkansas does not publicly provide more detailed information about CWD, we feel our proxy of a CWD gradient is justified and a realistic representation of the area, given the information that is publicly available about CWD in the region.

***The authors incorrectly conclude that an estimated stable population at one of the study sites results because CWD prevalence has reached stability. This conclusion is incorrect and needs to be reconsidered. Given the high prevalence typical of a stable equilibrium I don’t see why deer populations would remain stable? Finally, the authors should at least mention alternative hypotheses for population decline (harvest, predation, regional patterns) and absence of negative control (low/no CWD) in the study design. In particular, did increased harvest implemented as a management strategy cause population declines?***

We have adjusted our discussion to soften some of our stronger claims and discuss additional reasons that could have caused declines. Unfortunately, we have no information on predation or harvest effort for the state, but given that harvest limits have not changed in response to CWD, we feel that declines in harvest are likely correlated with declines in the underlying population. We hope that the additional discussion material will address many of the reviewer’s concerns.  ***Specific review comments:
The authors hypothesize that deer densities would decline across the study period due to CWD and therefore deer densities would be negatively associated with CWD prevalence. However, alternative reasons for potential population decline are not considered.***

We have added in an additional paragraph about the potential effects of predation. This paragraph now reads:

“While deer densities appeared to decline in response to high CWD sample prevalence in the region (Fig S1), additional stressors may be contributing to deer population declines. High predation rates from coyotes (*Canis lupus*) or black bears (*Ursus americanus)* may increase fawn mortality, limiting population growth (39,40). While coyote predation on healthy, adult white-tailed deer is relatively rare (41), reduced alertness in the later stages of CWD may elevate predator success and depress adult survival (42,4). However, given the high sample prevalence in both Newton and Searcy Counties during our study (maximum reported prevalence 50% and 18% respectively), it is unlikely that predation alone can explain the rapid declines in deer density. Rather, we hypothesize that declining deer densities reflect a negative population impacts of CWD.”

***Line 77-79: A CWD prevalence gradient was assumed based on a decline in the density of CWD detections in deer. While density of CWD detections could be a reliable surrogate for CWD this generally assumes similar spatial distributions of deer testing. Presentation of CWD prevalence data near the study sites would be a much better indicator of a prevalence gradient. I recommend the authors conduct a spatial analysis to quantify the landscape distribution of CWD for the study area to clarify and support their assumptions about spatial patterns.***

While we agree that a more in-depth analysis of CWD spatial patterns could be warranted in future studies, we were limited to the data that was made publicly available by the state. Based on publicly reported figures of known CWD positive cases, we believe the west to east gradient of CWD prevalence is a reasonable proxy for the true CWD prevalence in the area. However, we understand the reviewer’s concern about the spatial patterns and have added additional discussion material that acknowledges the assumed CWD gradient used in our study is only a proxy.

***Deer density was estimated using SCR methods and additional covariate modeling to account for distance from the initial CWD detection and the importance of pasture on deer density. I’m not an SCR expert so I can’t evaluate the technical components of this analysis. However, I had considerable difficulty integrating the different equations into a coherent framework. I strongly recommend providing a diagram to show how the various model parameters integrate and especially how they contribute to estimating density. At present I doubt that the current description would allow others to replicate the analysis.***

***I was especially concerned because I was never able to clearly see how “z” values were estimated and/or how they were used to estimate the precision of “N”.***

This is explained in Equation 3 (now equation 6), which shows that “z” is drawn from a Bernoulli distribution and the sum of all the ‘z’s’ represents N, the number of individuals in the population. This is typical for data augmentation approaches, which are used in many SCR type models in a Bayesian framework. We hope that our revisions to the methods sections make this approach more clear and easier for the reader to follow.

***Line 127: why did you choose 2 x 2 km pixels? Looking at Figure 3 there seems to be considerable spatial variation in density within each study site. I assume this spatial variation contributes to variation in “N”? It seems to me larger pixels would tend to smooth spatial variation and improve the precision of “N”. Are their guidelines for making this decision?***

We have added some additional details to explain our choice of the 2 km x 2 km pixel size. This section now reads, “We split the study area into 2-km by 2-km pixels to reflect the average summer home range size of white-tailed deer in the area” and includes a citation to a dissertation looking at home range sizes of deer in the study area.

***Lines 120-132: why do you assume the mean easting value represents the first detection of CWD instead of using the actual location? It seems you are assuming this location is the origin of CWD in this region (significant parameter in results)? Given the high prevalence upon discovery it seems highly unlikely this location happens to be the origin and/or center of the outbreak. You need to show both points on Figure 1 and clearly describe the rational and interpretation for this variable.***

Our description of this scaled variable was not very clear, so we have revised this sentence. We used the easting of each pixel in the model but scaled the variable, as easting values are quite large and unwieldly for estimation in this framework. The mean easting value does not represent the first detection of CWD in this area, but rather allows for the easting values to approximate a normal distribution for analysis.

***Line 134: it seems like your pasture variable is basically trying to remove the area of non-deer habitat?***

Previous research has shown that the presence of pasture lands can cause spatial variation in deer density. Therefore, we included this variable to help explain some of the spatial variation we saw in our deer detections.

***Line 148: not precisely clear what binary data for a day means: 0 for no deer, 1 for GE 1 deer (marked or unmarked)?***

By binary, we meant 0 for no deer, 1 for deer. We have clarified in the revised manuscript how the marked vs unmarked deer are used in the analysis.

***Lines 149-151: “k” needs to be defined. Is this a day? In general, I found some of your model parameters to be inconsistently/or not well defined. In addition, the units associated with the parameters need to be clearly defined.***

We have rewritten a large portion of the methods section to better clarify all the parameters and subscripts.

***Eq 3: How is “z” estimated – I found no equation for this variable? How was the precision of “N” estimated? Given the high variation of “N” in your figures you should make this clear.***

This comment is confusing, as this equation shows how z is defined. Equation 3 has two parts – z drawn from a Bernoulli with probability psi, and N as the sum of all the z’s. We are unsure what the reviewer means by this comment. Therefore, we made no changes.

***Lines 165-167: provide the time range when does tend to isolate. Why does July 1- July 14 maximize doe detection? How does this affect male estimates? Will many males have antler development by this time so that males and females can be distinguished – yearlings problematic? Why not use larger time periods to help increase precision of your estimates? This is mostly confusing with no particular rational or evaluation.***

Thank you for this suggestion. We wanted to maximum detection of all collared deer in our camera photos, while still being able to identify fawns and bucks. In our previous version, our sentence structure implied this time period only maximized female detections, which was misleading.

This section now reads, “To maximize detections of collared deer in camera photos, we chose to analyze deer densities from July 1 – July 14^th^ of each year, representing roughly 40 days after peak fawning (31). During this time frame, bucks, fawns and does are easily distinguishable from one another.”

We chose not to use a larger time frame as we wanted to ensure our population remained closed, which is an underlying assumption of this type of model.

***Table 1: It isn’t at all clear how alpha1 and alpha2 directly impact deer density. See comments about better description and integration of model components.***

We hope the revisions to the methods section have clarified how these variables contribute to estimating deer density.

***Table 2: Given the low encounter rates (and low precision) it isn’t surprising that estimated density also lacks precision? Can the encounter/detection rates be improved? Why is it important to present these model parameters, but not others found in Table 1? Will these estimates be useful to readers – how?***

These parameters are helpful for other researchers who are working on SMR models, as it can be hard to know what values are reasonable for deer and what values are a sign that the model is having trouble estimating parameters. While several of these parameters lack precision, they are similar to those published in other deer SMR model papers. In theory, future authors without any information on detection parameters could use these posterior estimates as priors for their own camera studies with unmarked deer.

Estimates for other parameters are found in the text.

***Lines 191-193: only deer with GPS collars used to estimate detection rates? Clarify how unmarked deer contributed to density estimates as well.***

We thank the reviewer for this suggestion. We have revised our methods section to make it clearer for the reader to follow our analysis and understand how each part fits together.

***Table 3: add how many detections of marked deer occurred.***

We thank the reviewer for this suggestion but disagree that it would be helpful information. Deer that stand in front of the camera for a long time, or have a home range that is centered near a camera trap will produce many detections, whereas deer that are farther away will not. As the parameters are jointly estimated across time, the total number of detections (mentioned in the text) as well as the estimates of lambda and sigma should give the reader a good idea of detection rates in this study. Therefore, we made no changes.

***Line 195: “CWD apparent prevalence in Newton County”***

We thank the reviewer for catching this typo.

***Lines 195-197: You should provide the annual prevalence data for these 2 counties. More importantly, you should provide the annual prevalence data surrounding your study sites. It seems like a spatial analysis of CWD detections is needed to understand the spatial pattern in detections/prevalence.***

We have included a supplemental table of apparent CWD prevalence in the counties surrounding our study sites. We do not have sufficient information for a spatial analysis of CWD detections as the reviewer suggested, as AGFC does not publish this information. However, we have included a graphic depicting the reported prevalence and hunter harvest for Newton County, Searcy County, Zone 2 and Gene Rush WMA based on publicly available information from AGFC.

***Lines 215-222: This short paragraph is the entire evaluation of the study hypothesis that CWD reduces deer density. It is crucial that statements made about deer density be rigorously justified using scientific/statistical analysis. Given the poor precision for density estimates inf Figs 2 & 3 simple statements based on median estimated density could be misleading. The study found a significant relationship for the easting parameter, but it isn’t clearly stated what this means – study sites further east had higher density? Statements in this paragraph about changes in deer density or density changes must be supported by statistical analysis that consider both median density estimates and their associated precision. Based on the high variation in estimated density I’m very skeptical that reported results are valid. Although the data is suggestive for the Gene Rush study site. I recommend that density changes over time be evaluated using trend analysis (regression) such that annual changes are statistically evaluated and quantified. Finally, the estimated deer densities in this paper are considerably lower than average deer densities in Arkansas (about 6 deer/km). Is this related to the study design? This needs some discussion about potential bias.***

We appreciate the reviewer’s comment and agree that a regression is necessary. To that end, we have re-run our analysis to include a time trend on expected abundance. We have included this new information in a new table (Table 4) and have adjusted our discussion to reflect these news results. All sites showed significant declines (as demonstrated by time trend estimates not including 0) except for female densities at Gene Rush. We believe this addition has made our statistical analysis much stronger.

We are unsure of the source for the ‘average deer density’ statistic that the reviewer mentions. Arkansas includes several distinct biomes, with high deer densities closer to the southern edge of the state. In the Ozark region where our study area is located, deer densities are limited by fewer food resources, higher human population densities, and more difficult topography. Thus, it is not surprising that our estimates are lower than the average deer densities for the state.

***Lines 231-235: it isn’t clear how this paragraph contributes to the paper. Given the lack of precision in estimated density it not surprising that density of males or females did not significantly decline. Please provide estimated precision for % female abundance. It seems surprising that female density would be lower than male density – as most deer populations are the opposite due to male harvest?***

This paragraph has changed significantly since our initial submission and we believe many of these concerns are no longer present.

***Line 242: this statement needs to be supported by a statistical analysis!***

We added in a qualifier that declines were at 3 sites, not necessarily the entire CWD zone.

***Line 248: minimal hunting but substantial predation***

We disagree with this assertion. The authors found that survival among uninfected deer approximated the range-wide average for adult mule deer. Thus, while the declines in their study could have been exacerbated by high predation, this does not explain the reason for declines at this location and not others. The sentence now reads,

“In Colorado, Miller et. al (4) documented an apparent 45% decline in mule deer abundance across a 20-year time frame when CWD prevalence was approximately 25%, despite the presence of suitable habitat, minimal hunting pressure, and survival rates in CWD-negative adults similar to those found elsewhere in the range.”

***Line 249: suggest saying 10% annual decline instead of .9 growth rate***

We have made the suggested change.

***Lines 253-269: this paragraph is important because it raises alternative hypotheses about why deer density may also change at the study sited. First, study lacks a negative control (low or no CWD) so trends at Post National are important. This area report about 10% annual decline in deer density over the similar time period. Could regional deer density be declining due to causes other than CWD (predation, harvest, etc.)? Also why are deer densities so much higher than your study sites? Harvest records in this paragraph are also useful because they demonstrate changes in harvest rates that could also influence annual deer density. Are some of the patterns you see affected by deer harvest? How can this factor be considered in your evaluation?***

We have added in an additional discussion paragraph to address predation or other causes for declines. We hope this change strengthens the paper and addresses the concerns of the reviewer.

***Finally, the fact that annual hunter harvest exceeds your density estimates makes me question whether your estimates are valid. You need explain this discrepancy.***

We thank the reviewer for catching this. We reported deer harvest in deer per square mile, but our own estimates were in deer per square kilometer. This made it confusing to read and made it appear as though harvest rates were greater than the densities observed. We have modified the paragraph to report estimates in square kilometers and have added in a supplemental figure showing trends in hunter harvest.

***Lines 275-281: you have likely misinterpreted Samuel (2023). There is no connection between stable equilibrium and deer density. Density is likely to decline with the high prevalence at Stage 4; however, this decline is independent of transmission dynamics and related to high prevalence. So, reaching Stage 4 does not mean that populations will become stable. Stable levels of infection are based on the population proportions that are infected, survival rates, birth rates of uninfected fawns, and transmission rates and not on deer density. Stage 4 can occur at different population densities because transmission is not density dependent. In summary, reaching stable CWD prevalence is not a sufficient reason for population stability.***

We thank the reviewer for correcting our citation. While we disagree with the idea that CWD transmission is not density dependent (this remains an open question in the literature), our use of Samuel 2023 was an incorrect citation to support our discussion points. We adjusted the sentence that uses this citation to now read: “However, as prevalence rises, densities may decline substantially.” Throughout this paragraph we have further avoided the phrase “enzootic equilibrium.”

***Lines 284-292: the paper provides a discussion and interpretation of the pasture parameter (alpha1). However, there is no similar discussion of the distance parameter (alpha 2) which seems much more relevant to the CWD-deer density hypothesis. How should alpha2 be interpreted relative to deer density and CWD distribution?***

We have adjusted our analysis so that the alpha parameters are easier to interpret.

***Figure 1: please add study site and county names to this figure. You should also add the first CWD detection and the mean easting used to calculate “easting” values. Providing an assumed gradient for CWD landscape distribution seems rather arbitrary. As recommended above a spatial analysis of CWD detection/prevalence would be preferred.***

We have added the study site and county names as suggested. The first CWD detection is somewhere 12 km to the west of Erbie, which would be off the map. However, we have added this information to the text. The mean easting value is not important, as it was just used to scale the easting variable and turn it into a roughly normal distribution.

***Figure 2: use “Estimated Deer…..”***

We thank the reviewer for their suggestion, but we have opted not to make this change. It is clear, given the error bars and the context of the paper, that these are estimated deer densities.

***Figure 3: please add study site and county names to the figure. Label should be “Estimated Deer Density”***

We have added the study site names to the figures, though we did not include the county names as they are listed in Figure 1 and would make it harder to see the rest of the figure.

***Figure 4: use “Estimated Deer ….”***

See response above

***Reviewer #2: 
Major comments: Figure 2 (and related text and conclusions), the error bars for the deer density data are extremely large and often overlapping in these data sets. I could not find any statistics on these comparisons, but aside from the starting density and ending density in the Gene Rush regions I doubt any of these values are statistically different. Did you apply any statistical tests to the data beyond error bars? Efforts to interpret this data should be expanded, even if the statistics suggest the population densities are not changing in two of the areas that should be made clear.***

We agree that the original analysis was imprecise and needed a statistical comparison to evaluate if the trend were present. We have re-run our analysis to include a time trend, which we believe has greatly improved our manuscript and should address the comments made by the reviewer. ***Conclusions in lines 250-252 and 30-31 would benefit from some mathematical modelling or more detailed numbers to back up the claim that populations cannot be sustained under the current regulations. This may in fact be true, but it is impossible to assess with the limited data currently shown. It is not clear how much hunter harvest is actually occurring in these areas compared to other sources of depredation and disease. If hunter harvest is a very small percentage of the deer removed, then perhaps the population won't be as affected? For example, if CWD is killing 13% of the population but hunter harvest is only 5% of the population then long term population effects will be much more dependent on CWD. On the contrary, if CWD is killing 13% yearly and hunters harvest is 35% yearly then I agree that the populations cannot sustain these additive pressures. However, without more specific numbers for the different sources of deer deaths (or % of the deer population) and the expected recruitment rate of fawns in these areas it is very hard to understand how these conclusions were drawn.***

Hunter harvest in deer populations is usually considered additive, especially when considered in the context of CWD. Even if CWD causes additional mortality beyond that of hunter harvest, adding harvest on top of CWD is likely to put total mortality above a level that could be offset by reproduction. Most studies of CWD suggest that annual adult survival for cervids with CWD is somewhere between 20-45%.

However, we have changed our wording to refer to population ‘viability’ rather than ‘sustainability’, as we would need more research to understand the role of hunters in driving (or offsetting) these population declines.  ***Minor comments:
Lines 26-28 and 220-222: Providing the final deer density values has little to no meaning to the reader in these locations as there are no starting values provided in the adjacent text to provide context.***

Thank you. We have removed this sentence, as it did not add anything to the manuscript. ***Figure 1: Label the three study regions by the names used elsewhere in the manuscript.***

Thank you. Great suggestion.  ***Can the authors comment on whether any major environmental effects or disease outbreaks occurred in the study regions during the study period, such as drought, wildfire, hurricanes, excessive rainfall during fawning season, EHD etc. that may have played a role? This seems especially important since there was discussion about an a unit in Benton County that experienced similar deer declines despite having minimal CWD (lines 257-259).***

This was a good suggestion and we added additional discussion of an extreme weather event that occurred in February 2021 and may have contributed to deer declines.

***Lines 264-267. The data on decreasing harvest numbers is helpful for discussion but would be stronger if you also included the hunter effort during these same years... If hunters declined you would anticipate harvest decline, and vice versa. Was hunter effort consistent? Was expected predation by non-humans fairly consistent?***

This is a great suggestion, but unfortunately AGFC does not measure hunter effort. The conclusion of this paragraph now reads, “While harvest trends do not always directly correspond to changes in abundance and may instead reflect changes in hunter effort (38), the observed trends suggest white-tailed deer abundance is likely decreasing both in the study area and across the broader CWD management zone.”

We also added an additional sentence summarizing harvest at Gene Rush WMA which may help add additional context to harvest trends in the area. This addition reads, “Additionally, harvest for Gene Rush WMA (located within Newton County) showed a steady decline from 1.53 deer per square kilometer during the 2020-2021 season to 0.85 deer per square kilometer during the 2024-2025 harvest season.”
