# Supplementary material for: White-tailed deer population declines in a high-prevalence chronic wasting disease region of Arkansas, USA
Source: PLoS One. 2026 Jan 7;21(1):e0340070. doi: 10.1371/journal.pone.0340070 (PMC12779150; doi:10.1371/journal.pone.0340070)
Supplement: Attachment — Submitted filename: Response_to_Reviewers_R2.docx [file pone.0340070.s006.docx]

Dear Dr. Caughey,

Thank you for giving us the opportunity to submit a revised draft of our manuscript, White-tailed deer population declines in a high-prevalence chronic wasting disease region of Arkansas, USA (Submission ID JWM-25-0070). We appreciate the insightful comments on our paper and have incorporated many of the suggested changes into our revised document.

Below, we detail our responses (normal font) to the reviewer comments ***(bold italics).***

***Reviewer #1 Summary Review:
I believe the paper as improved substantially from the first version. Improvements include better description of SCR methods, trend evaluation for deer density estimates, consideration of other potential factors affecting deer populations, additional data on CWD prevalence and harvest data.***

We thank reviewer #1 for their kind remarks.

***Overall, I believe some additional relatively minor improvements could be made to further improve the paper. I believe there is need for additional clarification of the SCR equations, in particular the time parameter (t). A sex and site-specific discussion of population trends would seem important with more specifics on estimation of the average annual trend and estimated CI – readers should be able to replicate your results.***

We have clarified the time parameter throughout the methods section. We also included additional discussion about the sex and site-specific population trends. We have also included CIs (in the supplemental materials) so that changes in the sex ratio can be evaluated from a quantitative perspective. The high levels of uncertainty inherent in unmarked SMR models make sex ratio comparisons largely uninformative, but we agree that this is good information for the readers to have.

***I have remaining concerns about the estimated sex-specific abundance results and trends relative to CWD prevalence. While adding a discussion of alternative population reduction mechanisms was helpful these could be reduced as they tend to distract from the larger results. The mention of density dependent transmission at the Erbie site is interesting and would benefit from further discussion/evaluation. This is an important aspect of CWD epizootiology that deserves more discussion. In general, I believe the paper would provide a stronger scientific contribution if it can generalize its findings relative to CWD management in both Arkansas and other CWD affected areas: harvest recommendations, CWD epizootiology, deer population decline from CWD, etc.***

Thank you, we have tried to address as many of the reviewer’s comments as possible within the manuscript. We have addressed specific points below, but overall we have included additional information about CWD transmission and an additional paragraph discussing the rate of decline seen in our population in comparison to other studies.

***Specific review comments:
Line 53: harvest limits***

We have changed the wording as suggested.

***Line 123: I believe you mean a starting date of July 2021?***

Yes, thank you. We started collecting data in November 2020 (collaring deer, etc) but did not analyze camera data until July 2021.

***Lines 122-124: consider moving this sentence to near 135-137?***

We moved the sentence as suggested.

***Lines 128-133: I still don’t like this somewhat arbitrary assumption about the distribution. Based on the Figure S1 it seems you could at least add that Newton County has much higher prevalence than Searcy – generally supporting your decision.***

We have added in a justification for this decision. This section now reads:

“Given the high CWD sample prevalence in Newton County relative to Searcy County (one county east) or Stone County (two counties east), we assumed a west to east gradient in CWD prevalence across the study area. To account for the presumed CWD gradient, we assigned each pixel an Easting value, which we scaled for analysis by subtracting the mean easting for the study area from the easting coordinate of each pixel and dividing by the standard deviation. We considered this a proxy for proximity to the first known CWD detection in Arkansas, as locations of positive detections in Arkansas were not publicly available.”

***Line 143 and later: the parameter t is not clearly defined. Later in the paper it seems to be defined as a 24 hr period between July 1 and July 14. Can you help the reader by giving better definition of t so it isn’t confused with annual change? Also, later in the paper you need to clarify if t is year-specific? If so it seems your equations with t would be year specific? For example, in EQ 1, if t is not year specific then how is it defined? Overall, I’m puzzled what specific time interval t represents. Also see comments below.***

We have changed the wording from “occasion *t*” to “year t” to clarify that *t* is a yearly subscript. In the context of the time-trend, this is a yearly trend. We are assuming that within the 2-week period of July 1- July 14^th^, abundance of deer does not change. This allows us to have up to 14 observations at each camera (max one observation per day), allowing for estimates of detection probability.

***Line 152: I believe you need to show a specific equation here – is this actually EQ 3? This is the crucial equation for estimating annual changes in density. Because this equation has a trend parameter it is not simply similar to EQ 1 and should be shown. Also, for time trends in Table 1 and later I suggest you use annual trends to distinguish from parameter t in your equations.***

The equation is identical for lambda and sigma, the only difference is the superscripts. These equations are defining the detection parameters, not abundance, so they are very different from equation 3. I don’t see the value in repeating the equation, since it is exactly the same format for sigma and lambda. Therefore, no changes were made.

***Table 1: The “a2” parameter is described as being relative to the first CWD detection, but your rebuttal comments indicated the first detection was west of Erbie – please clarify. It isn’t obvious why Erbie has a 0 prior on 2 parameters. Can you add a footnote to clarify this. For ‘a3” do you mean annual trend? Again, my confusion on t in your equations.***

We have changed the labeling to make this clearer. The label for ‘a2’ now reads ‘Relationship between abundance and Easting (proxy for CWD gradient)’. We have also changed our ‘a3’ label to say ‘annual trend’ instead of ‘time trend’ which was potentially unclear.

For the 2 parameters where Erbie is set to 0, this is because our equation also has an intercept. When working with categorical fixed effects, one can either have the fixed effect take on any value and remove the intercept, or you can include a global intercept and fix one category of the fixed effect to 0. We have chosen the latter option, using Erbie as our reference level.

***Line 165: please add the location for the easting point to Fig 1 so it is specifically defined. Without this location your results relative to easting can’t be replicated by others.***

We have added in the mean and standard deviation for the easting covariate in our results section so that our results are easier to replicate. The first paragraph of the results section now ends with the sentence, “To calculate the scaled Easting value that we used as a proxy for the CWD gradient, we used a mean value of 501979.4 and a standard deviation of 17070.23.” We also note that using the information provided in the data repository, this value can be easily calculated since we provide the boundaries of our study area.

***Line 169: I’m perplexed by this equation. Why is the “a3” part multiplied by (t-1)??? Do you mean evaluated at (t-1)? This part of EQ 3 is confusing and needs both clarification and explanation. If ‘a3’ represents a time trend (slope) how do you estimate first year (intercept? t=0?) sex and site-specific density? Also, I’m curious why you didn’t evaluate site-specific pasture parameters?***

The expected abundance in each year is based on an intercept (a0), a relationship to pasture (a1), a relationship to the presumed CWD gradient (easting, a2) and an effect of time (a3). In year 1, a3 drops out of the equation, since there’s no information before year 1. This formulation helps with identifiability.

We did not consider a site-specific pasture parameter, as we did not want to overparameterize the model. Unmarked data is only moderately informative and there is no information about any of the abundance covariates from the GPS data themselves (Stage 1). Thus, we tried to minimize extra parameters where possible.

***Line 174: is this the definition of t, a 24-hour period between July 1 and July 14? So, t is year specific and goes from 1-15 each year? Alternatively, t goes sequentially across 4 years 1-60? Or maybe t counts the elapse days since July 1, 2021? Again, I’m clearly missing something???***

See above for our definition of *t.* In this case, *t* goes from 1-4 and represents an annual time step. We have added in the phrase “year *t”* wherever we mention *t* in the manuscript to help clarify.

***Lines 200-204: Please cite a reference for this information here and in Fig S1.***

Thank you, we have added a citation to both locations.

***Table 2: in my view these are rather specialized parameters and could be reported in an appendix for interested parties.***

We have moved this to the supplemental information.

***Line 218: density was high at Grand River and Tyler Bend. This difference from Erbie deserves some discussion. Is it because of high CWD prevalence along your gradient? Does this provide further evidence of CWD impacts?***

Thank you for the suggestion. We discuss this later in the manuscript. The relevant section reads, “In our own study site, deer densities were lowest where presumed CWD prevalence was highest, but sex and site-specific time trends were not significantly different from 0. In contrast, deer declines were most severe at our most eastern site, Tyler Bend, with median deer densities declining more than 44% across the 4-year study and time trend estimates that did not include 0. These differences may be explained by the length of time CWD has been present at each of our study sites. Though not documented here, we theorize that our Erbie study site likely experienced similar declines prior to 2021 but is approaching an equilibrium between disease prevalence and density-dependent transmission as prevalence reaches high levels”

***Lines 222-225: I suggest you reverse these two ideas. First describe site and sex patterns both significant and not, as well as quantitative declines (with CIs). Then present the average decline (with CI) and describe how that average was calculated. Also, how the average (with CI) for each site was calculated for Fig 2. If that description is too technical it may need to be in the methods section?***

Great suggestion. We have added in a description in the Methods of how total abundance at each site was calculated and how we calculated the yearly declines. We also included the decline for each site. We opted not to change the order of the two paragraphs, but we incorporated the other ideas suggested by the reviewer.

***Table 4: Sex rather than Stage?***

We have changed the wording. Thank you.

***Lines 252-254: Given that 50% of your site/sex specific estimates seems to be equivocal (overlap 0) I think you should temper your statements here. In addition, this seems like a good place to discuss your more specific results and interpretation for site and sex differences. If your interpretation is that all sites are declining then some rational for that conclusion vs the typical 95% criteria should be concluded.***

While we have no statistically significant results to suggest that Erbie is declining (a3 median estimates were negative, but CI crossed 0), we found a significant decline in males at Gene Rush and both females/males at Tyler Bend. This suggests that both Gene Rush and Tyler Bend are declining (if females are stable at Gene Rush but males are in decline, then the net is a decline).

***Lines 254-263: seems like this comparison should be a new paragraph. Note that lions may have played an important part in the Colorado deer decline. Also, the declines you estimated for your study are considerably larger than the other studies you mention (or that I’m aware of). For example, a deer population would be reduced by 50% every 2 years using your results. No one has documented such a rapid decline and this deserves some discussion. Is part of your decline potentially due to harvest and/or factors?***

We have added in a new paragraph to discuss this. This section now reads, “The estimated declines in deer density in this region are some of the highest rates of declines ever reported for a cervid population with CWD. One plausible explanation is the high apparent prevalence (23%) in the region when CWD was first detected in Arkansas relative to initial prevalence estimates in other states (1-5% for Wisconsin, Colorado, and Pennsylvania). When CWD is detected early, targeted culling or adjustment of harvest limits may be effective at limiting population declines. However, once prevalence reaches high levels, management options become more limited and substantial declines are more likely. Our research underscores the importance of continued surveillance for CWD in areas without positive detections and the potential repercussions of delayed management actions. “

***Lines 261-286: I don’t disagree that the reductions you report suggest changes in harvest are needed. I wonder if you can make a stronger case for the hunting impact on decline and/or how much harvest needs to be reduced? Reduce male harvest, female harvest, both? Given the harvest data in Fig S1 can you approximate what proportion of the decline might be caused by harvest? Harvest data is covered in lines 272-280 but can you better integrate these with your results. Although CWD and harvest are surely partly compensatory you might be able to suggest how much harvest is viable?***

We appreciate this suggestion, but we believe we lack sufficient information to make this type of estimate. Harvest is variable between years, due to hunter success, hunter effort, and natural deer densities. We also have very little information about the areas of the county outside of our study areas. Thus, we feel there is too much uncertainty to make any stronger recommendations, though we do assert that harvest is likely too high.

***Lines 264-272: it seems to me that the 2 main messages here are that 1) deer density is much lower in the CWD area/your study area than many other parts of the state and 2) one other area has shown an unknown population decline not related to CWD. The later introduces the notion of other factors which follows. The first message brings up the potential that CWD may have different epizootiology and population implications based on deer density (see later comments).***

This comment suggests no specific changes.

***Lines 287-303: I’m glad you added this type of information, but I suggest it be condensed substantially. These are other possibilities that might contribute to population decline, but likely much less important than high CWD and/or harvest.***

We added in this information in response to another reviewer, who was concerned that these factors were potential mechanisms adding to population decline. While we personally agree that CWD and harvest are probably the main factors, we want to acknowledge that other drivers are possible. Therefore, we made no changes.

***Line 308: as prevalence reaches high levels***

We have made the suggested change

***Line 309: our Erbie study site***

We have made the suggested change

***Line 312: annual trend***

We have changed the wording as suggested.

***Line 313-318: I don’t believe that reference 50 shows DD transmission. Instead, I suggest you review Storm et al (2013; Ecosphere). This paper indicates that CWD is generally FD at higher deer densities but DD at low densities – as far as I know this is the only study with data that shows potential for DD transmission? I think your study areas would be characterized as low density relative to Wisconsin. Would this provide a justification for DD transmission in your areas, but perhaps other patterns in higher density areas – similar to Wisconsin (Samuel 2023)? In addition, DD transmission doesn’t produce a sable equilibrium as you suggest. It typically means high prevalence reduces population levels, which reduces transmission rate and prevalence, then populations increase and the cycle starts again. See Almberg’s 2011 modeling paper. I think your predictions should try to account for these predicted D transmission/population patterns. If you can pull all this together it could make a very useful contribution of how CWD works in low density populations.***

Thank you, we accidentally cited the wrong Samuel and Storm paper. We have corrected the citation. We have added to this paragraph additional information about the CWD density dependent cycle. We had previously used the term equilibrium rather loosely – implying the average density that the population will reach as it ebbs and flows following the density dependent cycle. This section now reads, “These differences may be explained both by the length of time CWD has been present at each of our study sites and possible density dependent transmission rates. Though not documented here, we theorize that our Erbie study site likely experienced similar declines prior to 2021 but is approaching a temporary equilibrium between disease prevalence and density-dependent transmission as prevalence reaches high levels. Previous models of CWD suggest that after initial population declines, local CWD prevalence and transmission rates may be lower due to low population densities. As the population reproduces and densities rise, prevalence increases and the cycle continues. Therefore, while our Erbie study site may show minimal declines in the short term, we predict future declines are possible once population densities rebound. Furthermore, we predict that population declines at both Gene Rush WMA and Tyler Bend will continue unless CWD prevalence is reduced or the population reaches lower densities.”

***Fig S1: please provide citation(s) for the data in this figure.***

We have added citations as suggested.

***Fig 1: please add the mean easting point to this figure. In my opinion your gradient is artificial and unnecessary. I think the county level prevalence data in Fig S1 is sufficient to show western areas have higher CWD. The easting point location is needed so others can replicate your analysis.***

We have included the mean and standard deviation of the scaled easting value in our results to allow others to replicate our analysis. The mean easting point would be a dot in the exact center of the map and would not add anything to the figure. I think there is a fundamental misunderstanding about how the easting value is used in our analysis, which was likely caused by our earlier draft which was not clear on our analysis. In our study, the easting value is simply a way to quantify the east-ness of the pixels in our study area. In it of itself, this value is not meaningful, but is used as a proxy to explain the spatial variation we see in the data. By including this gradient, we are able to estimate how moving to the east might change expected density of deer. We acknowledge that this is a proxy, but we believe our decision is justified by the sample prevalence reported by AGFC.

***Fig 2: does not require colors and could be done in BW with different markers for each study site.***

We have added in different marker shapes to allow the graph to be more easily read by color-blind readers. However, we believe the colors make it easier to distinguish the two high density sites from one another in the later years, so we have kept them.

***Fig 3: these detailed data are ancillary to the study and could be moved to supplementary material***

We acknowledge that this is true if the goal is simply to look at general trends, but since this was a large spatial analysis, it seems fitting to include a map of spatial trends. We have decided to keep it in the main manuscript.

***Fig 4: can be done in BW. I have remaining concerns about the data in these figures. First, most hunted deer populations have considerably higher female deer abundance than males due to selective harvest. For example, males are typically about 1/3 of the population. Do you have data that would suggest equal abundance for males and females is typical? Second, your first year estimates show equal density of males and females, followed by males appearing to decline faster than females. This suggests males are more “vulnerable” to CWD than females, which is quite consistent with the literature. However, given the apparently high CWD prevalence in Newton County, why hasn’t CWD already reduced male abundance compared to females in 2 of your study sites? Both of these results reduce my comfort level in your results. I think you should address these concerns in the discussion.***

We thank the reviewer for his suggestions. A major drawback to SMR models is a high level of accompanying uncertainty. While we attempted to estimate the changes in sex ratio over time, the uncertainty from the model made it difficult to reach any specific conclusions about the sex ratio of this population. While our results do appear to show initially ‘equal’ sex ratios, the 95% credible intervals suggest the population may have started at 60% female at Erbie and 58% female at Tyler Bend. We recognize these CIs were not previously mentioned in the text, so we have included them in a graph in the supplemental information (see Figure S1).

Additionally, harvest records from Gene Rush (the only site where we have specific harvest information) show that the male to female harvest ratio has been < 1 in 6 of the past 8 years. In 2019, the harvest ratio was exactly 1 (49 antlered, 49 does). In 2023-2024 (the final year of our study), the harvest ratio was 0.82 (31 antlered, 38 does). Assuming a roughly 50% sex ratio at birth, it is not surprising that the population sex ratio was close to 50%. While this information is interesting, we do not include this in the text as our focus was on the overall population, not specifically the buck to doe ratio in this area.

***Reviewer #2: Thank you for your efforts to improve your manuscript. The results and figures are much more clear now, providing a cohesive and easily interpreted message.***

We thank reviewer #2 for their comments.
